# Supplementary material for: Reversible bending of U-shaped plant petioles under dehydration
Source: Quant Plant Biol. 2025 Nov 12;6:e41. doi: 10.1017/qpb.2025.10030 (PMC12722063; doi:10.1017/qpb.2025.10030)
Supplement: Schliebach et al. supplementary material [file S2632882825100301sup001.zip › SupplementaryInformationV17.pdf]

Supplementary information

Anne Schliebach, Mohammad Nadim Kamar, Baptiste Bordet, Catherine Quilliet, Benjamin Dollet, Eric Badel, Emmanuel Siéfert and Philippe Marmottant

October 16, 2025

Supplementary movies

- Movie M0: A short movie clip summarizing all findings can be found at the url: `https://youtu.be/v40KD5OH1QM`
- Movie M1: Spathiphyllum plant drying and then rewatered
- Movie M2: Plant not watered during 20 days and then rewatered
- Movie M3: Opening of the U-shape and folding

Supplementary figures

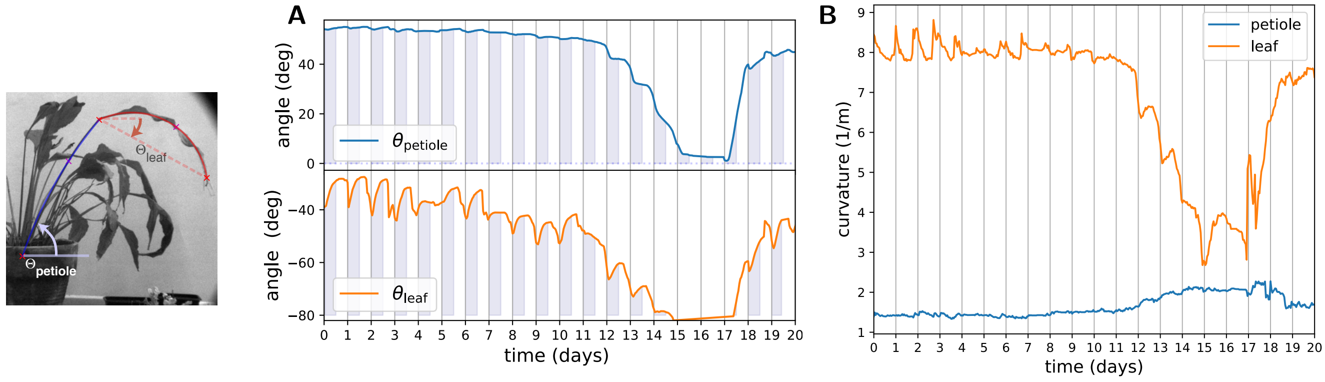

Fig S1: (A) Evolution of the angle of the petiole (top) and the leaf (bottom), t=0 at 5 pm showing the daily cycle of leaves falling during the day then rising during the night (B). Evolution of the global curvatures of the petiole and the leaf, whose shape is fitted by an arc of circle. While the petiole slightly curves, the leaf straightens when falling and aligning to the the vertical.

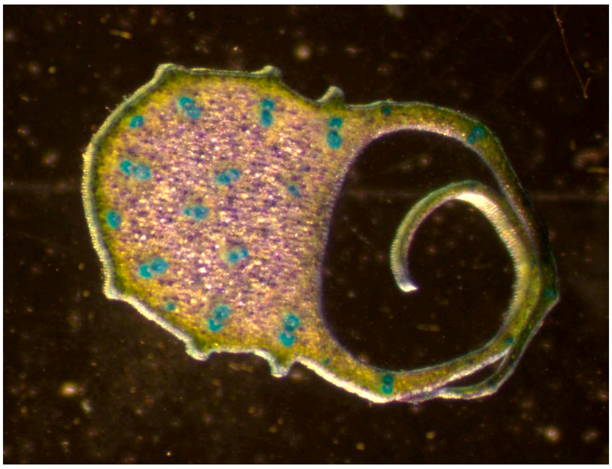

Fig S2: Transversal cross section of the petiole stained by immersion during 30s in a solution of toluidine blue. Blue zones enlighten the lignified cell walls. Red zones enlighten the cellulose

.

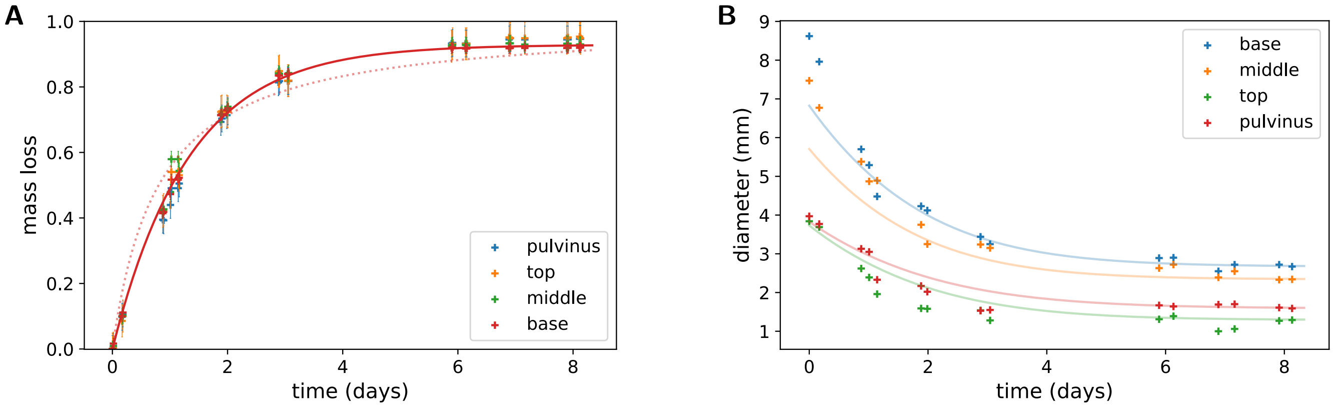

Fig S3: Mass loss (A) and diameter (B) for cut pieces of the petiole, with length from junction to base $L = 4.9, 4.1, 3.7, 3.8$ cm. The continuous line is a fit with a decaying diameter and evaporation flux $k = 1.8, 1.7, 1.6, 1.6 \times 10^{-4}$ kg/s/m^2, for the pulvinus, top, middle and base respectively. Model for the diameter: assuming that water mass is transformed in volume (density ρ of water), and that total volume is $V = \frac{\pi}{4}d^2L$ with L constant gives $d = [d_{\text{dry}}^2 + 4(M - M_{\text{dry}})/(\pi L \rho)]^{1/2}$ with d_{dry} the final dry diameter, M the mass and M_{dry} the final mass. This model works well for top and pulvinus diameters, but not for base and middle diameters, probably because the shape of the cross section is not round.

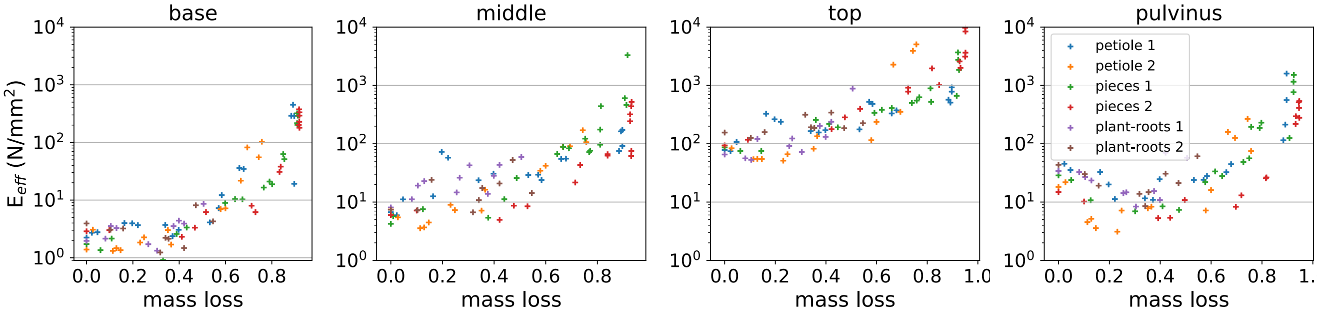

Fig S4. Effective Young's modulus E_{eff}, computed from the flexural modulus $B = E_{\text{eff}} I$, with $I = \pi d_{side}^4/64$; i.e. assuming a homogeneous cylinder of diameter d_{side}, the diameter measured from the side with a caliper. Here we plot $E_{\text{eff}} = B/I$. Note that E_{eff} looks constant until a mass loss around 0.4.

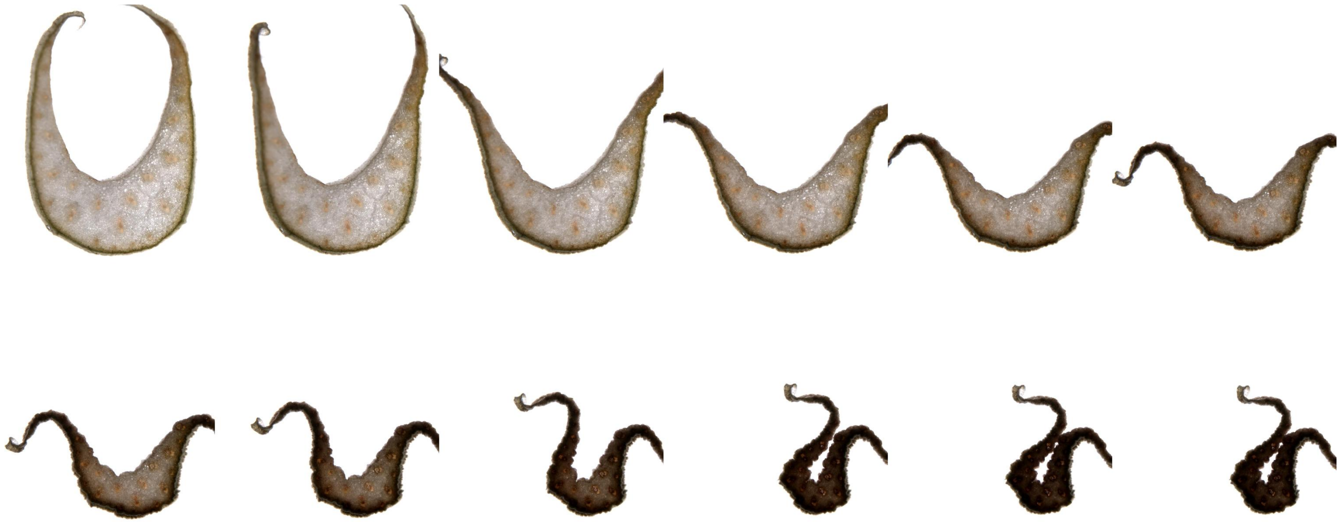

(a)

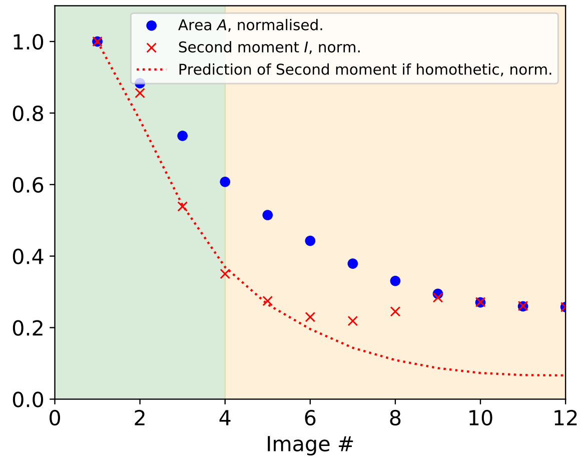

(b)

Fig S5. (a) Complete drying of a cut slice of the U-shaped base, with an image every 10 min, starting from top left image. The initial width of the plant section is 13 mm. (b) Evolution of the area of the cross-section area A (circles), and second moment of inertia I (crosses), and predicted I for an geometrically similar evolution (dotted line), normalized by their initial value. The prediction value of the second moment of inertia assumes a geometrically similar change in area but not in shape, with variation as $I_{\text{pred}} \sim d^4$, with d such that $A = \pi d^2/4$. The green area is the reversible range for loss of area less than 40%, the orange area is the non-reversible region.

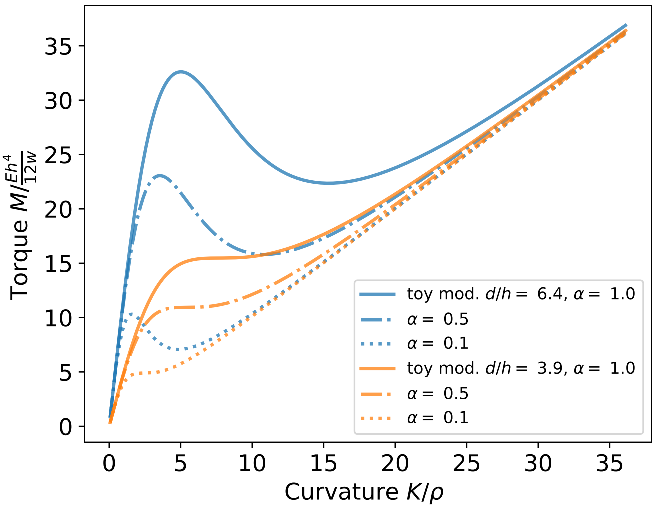

Fig. S6. Effect of anisotropy on the toy model for various non-isotropic values α and various d/h values.
