# Supplementary figures and images for: Reversible bending of U-shaped plant petioles under dehydration
Source: Quant Plant Biol. 2025 Nov 12;6:e41. doi: 10.1017/qpb.2025.10030 (PMC12722063; doi:10.1017/qpb.2025.10030)

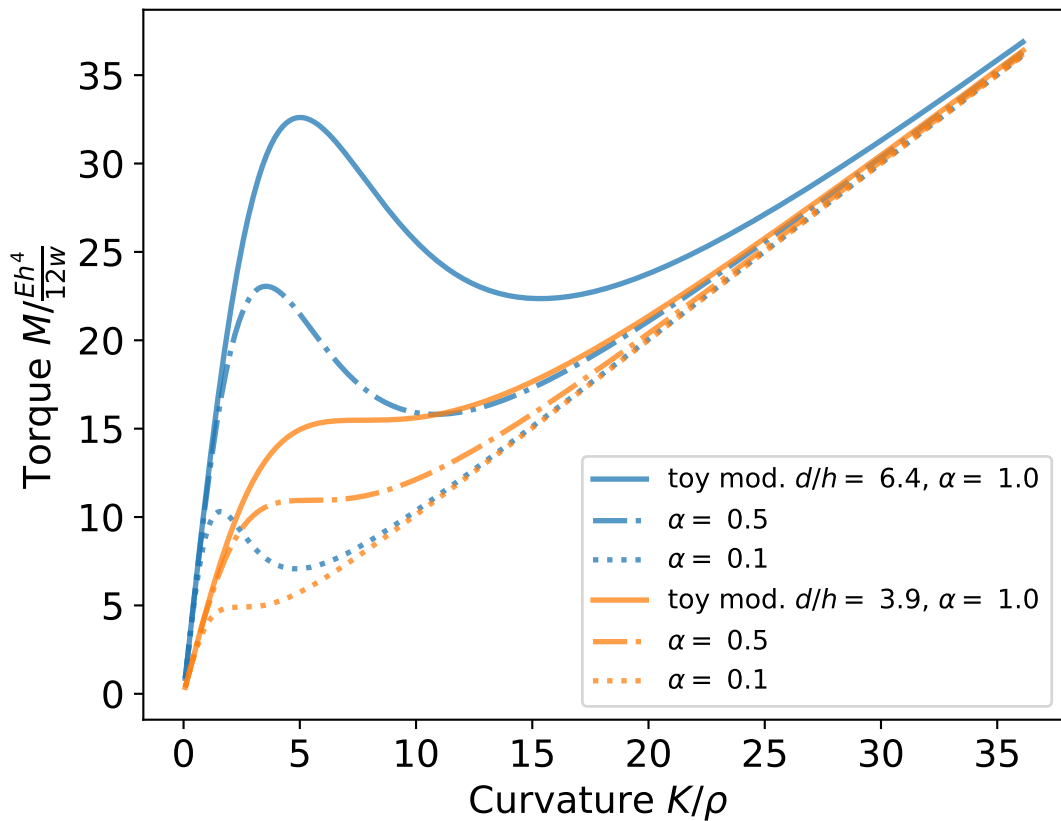

Supplement: Schliebach et al. supplementary material [file S2632882825100301sup001.zip › FigS6_BucklingTape_Non_Isotropic_Article_alphaBF.pdf]

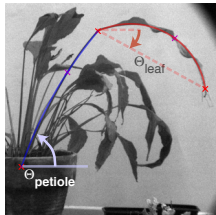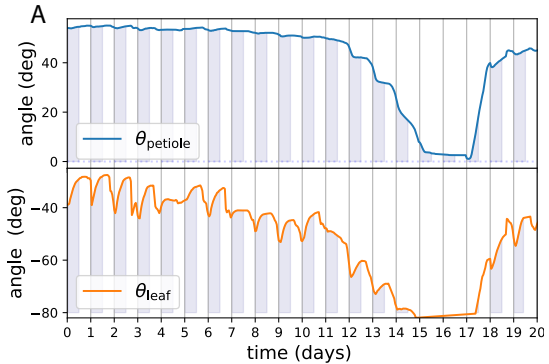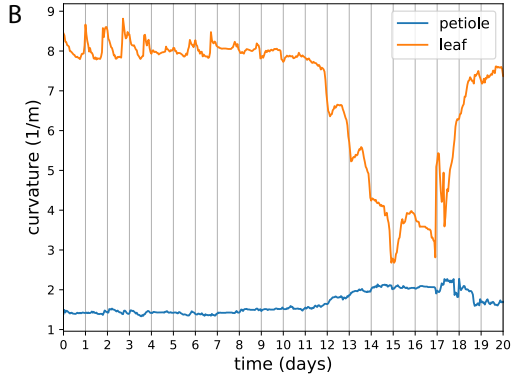

Supplement: Schliebach et al. supplementary material [file S2632882825100301sup001.zip › FigS1.pdf]

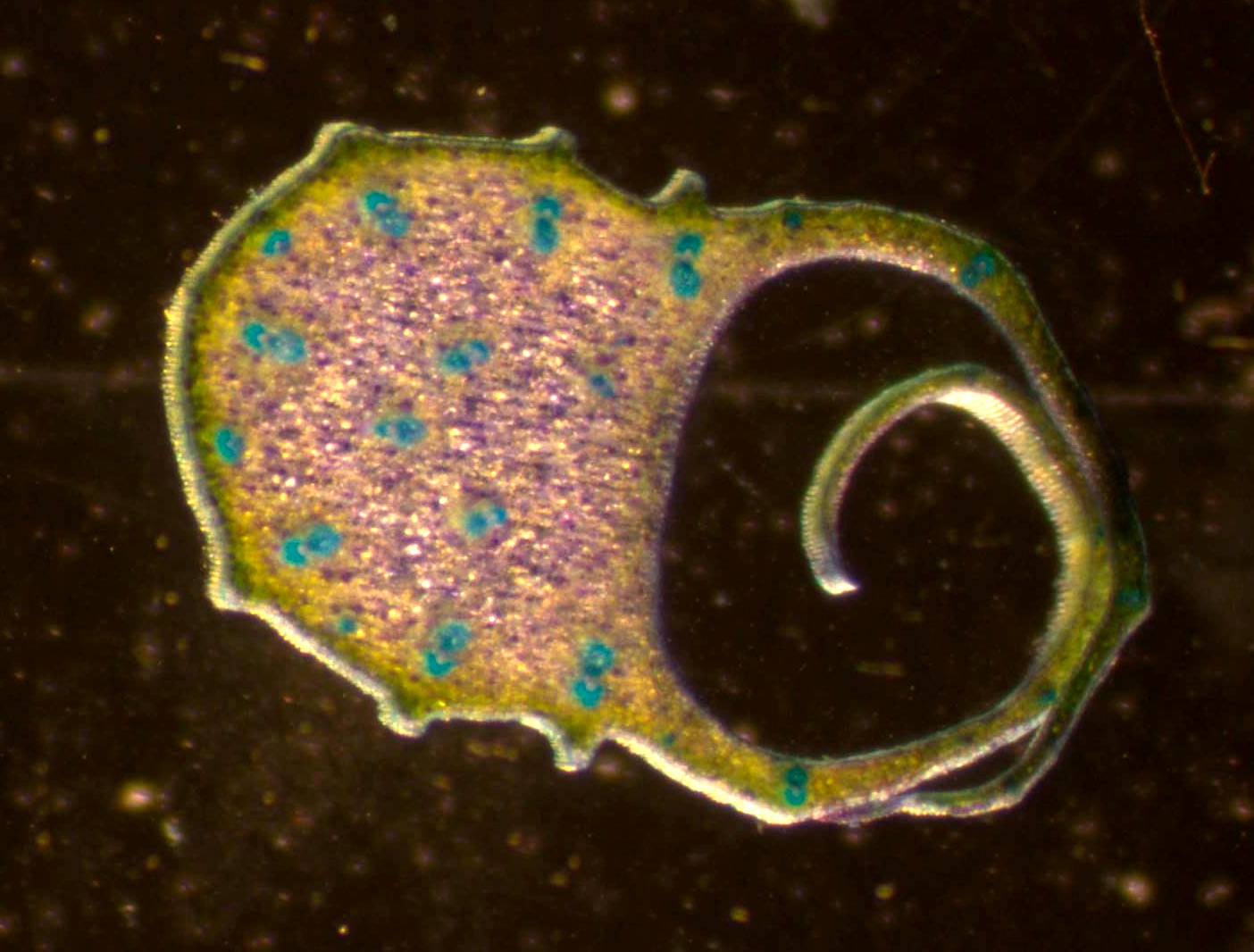

Supplement: Schliebach et al. supplementary material [file S2632882825100301sup001.zip › FigS2coupe_milieu-1_enhanced-cropped-Flora.jpg]

A

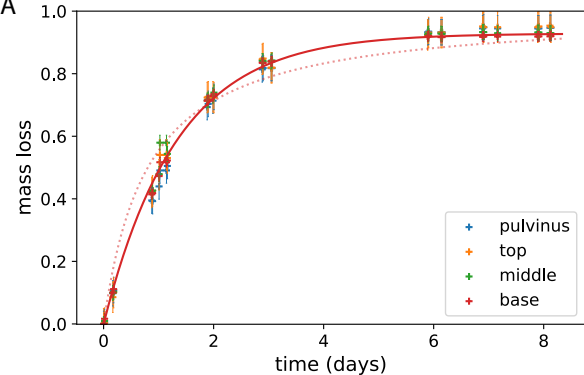

B

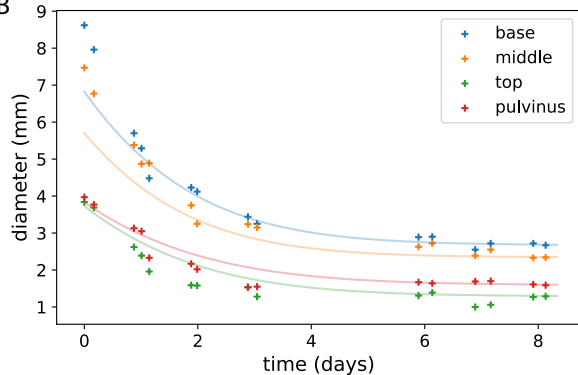

Supplement: Schliebach et al. supplementary material [file S2632882825100301sup001.zip › FigS3.pdf]

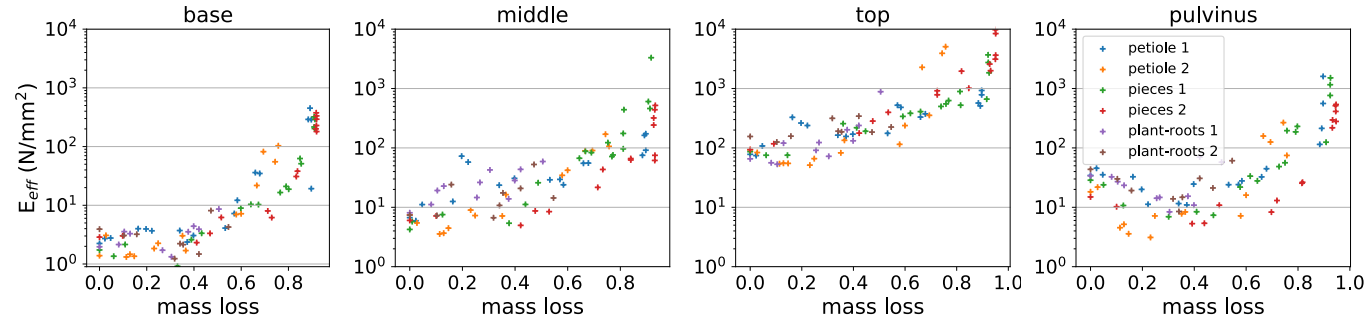

Supplement: Schliebach et al. supplementary material [file S2632882825100301sup001.zip › FigS4.pdf]

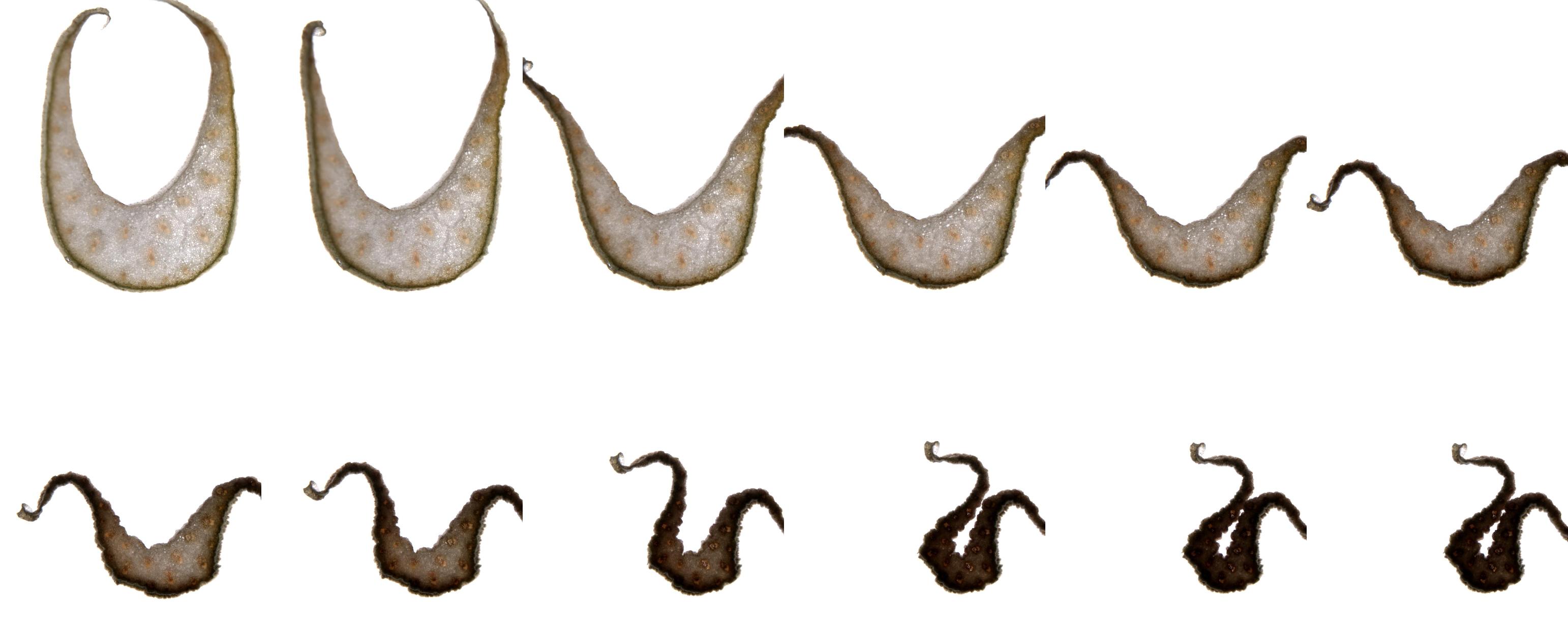

Supplement: Schliebach et al. supplementary material [file S2632882825100301sup001.zip › FigS5_Montage_Movie1_scale0dot06muperpix.jpg]
